# What to Send to the Microscopist and How to Prepare It

**Published:** 1900-07

**Authors:** T. E. Oertel

**Affiliations:** Professor of Pathology and Bacteriology; Medical Department of the University of Georgia, Augusta, Ga.


ATLANTA

### Journal-Record of Medicine.

Successor to Atlanta Medical and Surgical Journal, Established 1855, and Southern Medical Record, Established 1870.

VOL. II. JULY, 1900. No. 4.

EDITORS { BERNARD WOLFF, M.D., DUNBAR ROY, A.B., M.D.

M. B. HUTCHINS, M.D., BUSINESS MANAGER.

PUBLISHED MONTHLY. OFFICE 305 AND 306 FITTEN BUILDING.

### *ORIGINAL COMMUNICATIONS.*

### WHAT TO SEND TO THE MICROSCOPIST AND HOW TO PREPARE IT.

BY T. E. OERTEL, M.D.

Professor of Pathology and Bacteriology; Medical Department of the University of Georgia, Augusta, Ga.

Within comparatively a few years the microscope has become a valuable, and in many cases, an indispensable ally to the general practitioner.

Many of us can remember when the etiology of some of the most common and yet most fatal diseases was but half understood or shrouded in the darkness of ignorance.

It is now almost difficult to believe, that not until 1882 was the causative agent of tuberculosis discovered, and that the ordinary pus organisms were not isolated until 1884, when Rosenbach succeeded in growing them upon artificial media and demonstrated their relation to suppurative processes and thus laid the foundation for modern aseptic surgery.

There is not a department of medicine which has not made immense forward strides during the past twenty-five years, and an analysis of the subject will show that the microscope is directly responsible for the greater part of this advance.

The result is that in our schools the subject of microscopy in all its branches is yearly receiving more attention. The larger universities have each spent hundreds of thousands of dollars upon laboratories fitted with all the necessary facilities for microscopic research.

Thousands of eager workers are toiling at this new bench of industry and freely giving to the profession at large, and thus to the world, the results of their labors.

The literature of the subject has become enormous, and in order to become expert in the science, one must devote years to patient and industrious work.

The general practitioner who was graduated some years ago, when microscopy in the schools was in its infancy, has in most instances, perforce, not kept up with this particular line of work, and probably his practice has prevented him from going to the medical centers for the purpose of especial study in the microscopical laboratory; or he has felt that the time he could devote to it would not warrent him in the necessary expenditure of time and money. Even if he has purchased a microscope and is competent to do the more ordinary work of urine and sputum examination, he has neither the equipment nor the knowledge necessary to determine the forms of tumors or the pathology of tissues. Despite this, if he be abreast of the times he must often feel the need of the aid the microscope can give him, and turn to his brother, who has special training in the microscopical laboratory, for help and guidance. But even here he is confronted by difficulties. The technique of the laboratory is exacting, and the methods laid down in the text-books on the subject are so numerous as to be confusing. How shall he prepare the material he wishes to send to the laboratory, or, what is of as much importance, how shall he not prepare it?

It is with a view to elucidate this matter and to furnish a concise guide as to the selection of materials for microscopical investigation and the simplest mode of preserving them for transmission to the laboratory that this article is written.

TUMORS.

In every case where there is room for doubt as to whether a neoplasm is benign or malignant, the value of a microscopic examination, prior to operation, is evident, for by it we are able to ascertain the exact nature of the growth and thus to better judge of the steps that should be taken for its removal.

It is often of the greatest moment that we should know whether a given area of ulceration of the cervix is a simple erosion or the result of a carcinomatous nodule. The same may be said of neoplasms elsewhere, and often we are able by the use of the microscope to detect, absolutely, the character of a tumor long before it has reached a stage of growth which would render possible a positive diagnosis by the ordinary clinical methods.

Cutaneous growths which are supposed to be carcinomatous, those tumors of the mouth so often sarcomatous and classed under the general term ranula, neoplasms of the nasal and post-nasal passages, all of these may be diagnosticated at the expense of little inconvenience to the patient and a few days' time.

Of course it is necessary that a piece of tumor should be excised, but it need not be large, nor is a general anesthetic indicated in other than exceptional cases. One should cut out a piece about a quarter of an inch square, being certain that it comes from the new growth, and this should be put directly into at least one ounce of strong alcohol without being previously washed in water, neither should it be handled more than necessary.

Commercial alcohol is supposed to be 95 per cent. strength, but generally falls somewhat short of this. If absolute alcohol can be obtained, so much the better, and of course it should be used undiluted. The tissue may now be sent to the laboratory in a bottle filled to the cork with alcohol, as this prevents the possible damage of the softer structure during rough handling.

The location of the neoplasm, its size, the rapidity of its growth, and any general data bearing upon it should always be noted and the information sent with the specimen in all cases.

TISSUES IN GENERAL.

Tissues procured by autopsy may be preserved in any one of the fixing agents in common use; formulæ for which will be found at the end of this article.

If possible the autopsy should be held within six hours after death, in order to guard against post-mortem changes. In cold weather such changes may not take place for several honrs longer, but under all circumstances it is better if we can obtain the tissues in as near the recent state as possible.

Small portions of the various organs—lung, liver, spleen and the like—should be put directly into the fixing agent.

If the fluid selected be alcohol, the pieces should not be more than a quarter of an inch thick, though they may be somewhat longer and wider, except in the case of lung tissue. Unless lung is solidified it is almost impossible to cut it so thin without crushing it and causing it to collapse. The nature of lung structure admits of the more ready penetration of the fixing agent, and we may therefore cut pieces half an inch in thickness and still secure good fixation with alcohol.

Sections of all tissues should be made with a very sharp knife, and at one stroke. A razor is perhaps the best tool, if one has not a knife made especially for the purpose.

If Müller's fluid or Zenker's fluid be used, the pieces of denser organs may be as thick as half an inch. If, on the other, bichloride of mercury solution be used, the pieces should never be larger than one-quarter of an inch square, as it does not penetrate well.

BLOOD.

The microscopic examination of blood is of especial importance in cases of suspected malaria, anemia, leukemia, trichinosis and septic and pyemic conditions. In many cases a blood count is desirable, but this is, of course, impossible unless the microscope can be brought to the bedside. Smear preparations must be made, and with a little practice this is a simple procedure. These keep indefinitely and serve for the examinations for malarial parasites, bacteria and for differential counts of white blood-corpuscles. Whether the spread be made upon the cover-glass or glass slip, the two essential features that are necessary for a successful smear are absolute cleanliness of the glass and a drop of blood of the proper size. The beginner is apt to make the smear too thick. The usual method of procedure is as follows:

Clean two cover-glasses ($\frac{7}{8}$ squares, No. 2, are the most suitable) with soap and water in order to remove any trace of grease, polish each with a soft, clean cloth, and this must be repeated at the bedside of the patient; clean the lobe of the ear with soap and water, make a free puncture in the lower part of the lobe, wipe away the first few drops that flow, and then catch a fresh drop of medium size upon one of the cover-glasses preferably held in a forceps; quickly invert it and bring it in contact with the other cover-glass, allowing the edges to slightly overlap. The blood should spread evenly between the two surfaces and the glasses should then be pulled apart by *sliding* one from the other. If correctly done this should give a thin, even smear on each cover-glass. They are allowed to dry in the air and then may be kept for months, and, if protected from dirt and moisture, will not deteriorate.

Smears may be made upon the glass slips by catching a drop of blood on the end of one and with the slip slightly inclined drawing this end across the surface of another slip which has been properly cleaned.

A micro-chemical test may be made of stains which are suspected of being blood stains, and it may thus be definitely ascertained whether blood be present or not. The article upon which the stain is, may be sent intact or the stained portion cut out, if it be a garment. Stains upon walls and floors or pieces of furniture may be excised and sent to the laboratory, care being taken in forensic cases that this be done in the presence of witnesses, that there may be no question as to the identity of the stains examined.

### SPUTUM.

Much may be learned from careful microscopic examination of sputum. Among the elements of great diagnostic value may be mentioned elastic fibers, indicating a destructive process in the lungs, spirals, fibrinous casts of the smaller bronchi molds, which may at times be pathogenic in the lungs, actinomyces, echinococcus cysts and hooklets, crystals and bacilli. Among the latter the tubercle bacillus is by far the most important.

It would seem at this day quite unnecessary to urge microscopic examination of sputum, in every case in which there is the slightest ground to suspect the presence of tubercular lesions.

It is however a lamentable fact, that the profession at large seems either blind or indifferent to the fact that in this class of cases the microscope is not only the most valuable guide to correct diagnosis at its disposal, but far more than this, that the duty of the physician to his patient makes it obligatory.

There is still that higher and more important duty to the community at large to discover as soon as possible every case of pulmonary tuberculosis and see to it that the patient be instructed as to the proper care of his sputum, so that he be not a menace to the happiness and lives of his relatives, friends and the public at large.

In tubercular cases, the sputa first raised in the morning is more apt to contain the bacilli.

It is not necessary to add any preservative to sputum, as it will keep for several days and the B. tuberculosis may be found if present even after putrefaction and liquefaction have taken place. One may add a few drops of carbolic acid in case only an examination for B. tuberculosis is to be made, as this enhances the staining qualities of the organism and acts as an antiseptic as well. If animal inoculation is desired, or cultures are to be made, of course, this is not permissible.

### URINE.

This may best be preserved by adding a drachm or so of chloroform to a four-ounce bottle of urine. It is better to collect the urine passed about two hours after a hearty meal.

If it is desired to make a quantitative analysis, the total quantity passed in twenty-four hours must be collected and measured and a mixed sample of this sent to the laboratory together with data as to the total amount passed.

The urine may be examined for tubercle bacilli in suspected tubercular disease of the genito-urinary tract. If this is desired, it is well to collect a considerable quantity of urine, allow it to stand for about ten hours, and decant the clear, upper portion, reserving the lower, in which will be the sediment, for examination. If the microscope fails to reveal the tubercle bacillus, it may be that an animal inoculation may prove its presence.

### VOMIT.

A minute examination of vomited material will often aid in the diagnosis of gastric ulcer, chronic gastritis and cancer of the stomach. Pus has also been found in vomit, indicating a suppurative process of the gastric wall or a rupture in the gastric cavity of a neighboring abscess.

The addition to vomit of a small quantity of chloroform will prevent decomposition and will not interfere with the future investigation of its solid constituents.

### FECES.

Fecal matter may contain various intestinal parasites and their ova, tubercle and other bacilli, blood, pus, portions of undigested food, etc. It is best to send it without adding a preservative, provided the delay in transmission is not too great.

Of course, in a bacteriological examination by culture, or if animal inoculation is desired, the material must be collected in sterile vessels and thorough aseptic precautions taken in its subsequent manipulation.

### CRUSTS AND SCALES.

Crusts and scales from cutaneous diseases may be sent dry.

An examination of these may reveal the nature of the malady if it be due to some cutaneous parasite.

The mistake, which is quite common, of sending the crusts from a supposed epithelioma with a request for information as to whether or not such is the case, should be avoided, as a piece of tissue only is of value.

### HAIRS.

Hairs may be examined to determine a pathological condition, such as the forms of fungi which frequently attack them, and also for medico-legal purposes. In either case they should not be submitted to the action of any reagent.

It is best to put them in a small clean bottle. If they are from a forensic case they should be collected and placed in the bottle in the presence of witnesses, and the bottle sealed and marked with a distinguishing sign, in order that there may be in the future no possible question as to the identity of the hairs examined. The hairs of many of the lower animals are very different in structure from human hair, and it may be a simple matter to prove that hairs found upon an instrument supposedly used in committing murder are not human hairs, which would, of course, be a strong point in favor of the defense.

PUS.

Pus from various situations may be examined for pyogenic organisms, actinomyces, anthrax, B. tetani, tubercle bacilli and gonococci. If other than a microscopic examination is desired, of course the material should be collected and handled under aseptic precautions, and no reagent added.

It is generally very difficult to demonstrate the tubercle bacillus in pus from an abscess or tubercular joint except by animal inoculation. This consists in injecting subcutaneously or into the peritoneal cavity of a guinea-pig some of the suspected material. The animal is allowed to live five or six weeks, when if the injected material was tuberculous the characteristic lesions will be found in the animal.

The secretions of the urethra in cases suspected to be due to the diplococcus of Neisser may be positively diagnosticated by means of the microscope.

Particularly is this of value when one who has recently had gonorrhea consults the physician relative to the propriety of his marriage. It is well known that such advice is frequently sought, and if the patient is allowed to marry while there are still present in his urethra crypts the organisms of gonorrhea, the physician may be responsible for the unhappiness or even the death of an innocent woman, who might have been protected had he done his full duty. High authorities do not hesitate to assert that *all* cases of pyosalpinx are due to the above organism.

Long after the specific urethritis has subsided we may be able to find the gonococcus in the mucous threads common to the urine of such cases. If necessary a discharge can be set up by the use of stimulating injections or the free drinking of beer.

Smears of the pus should be made upon the glass slips sold for microscopical purposes, or, if these are not at hand, upon strips of window-glass, or the pus may be collected in a vial, or even, as a last resort, upon a piece of cloth, or the stained dressings may be sent to the laboratory.

Several examinations may be necessary before a positive conclusion is arrived at, but who can deny that any amount of care and toil and expense are better than the slaughter of the innocent which daily takes place from this cause.

### CYSTS.

Examination of cystic fluid obtained by aspiration sometimes leads to the determination of the cause of the pathological process.

Hooklets and other portions of echinococcus cysts may be found in hydatids.

The addition of chloroform in small quantities will act as a preservative.

### SEMEN.

In rape cases the contents of the vagina obtained by wiping it out with a piece of clean gauze may show the presence of spermatozoa. These may exist in the vagina for several days.

Any stains upon the garments should be examined also in order to determine their nature.

The whole garment or the stained portion alone may be sent to the laboratory, the usual precautions as to the identity of specimens in medico-legal cases having been taken.

### FORMULÆ.

*Muller's Fluid.*

| | |
|---|---|
| Bichromate of potassium - - - - | 1 part. |
| Sulphate of sodium - - - - | 2.5 " |
| Water - - - - - - - | 100 " |

One of the best general fixing agents. About twenty times the bulk of the tissue should be used and the fluid changed as often as it becomes turbid, which will be every day for several days.

*Zenker's Fluid.*

| | |
|---|---|
| Bichromate of potassium, - - - | 25.00 |
| Sulphate of sodium, - - - - - | 10.00 |
| Bichloride of mercury, - - - - | 50.00 |
| Water - - - - - - | 1,000.00 c. c. |

Before using add 1 c. c. of glacial acetic acid to each 20 c. c. of the mixture. This solution is much used and is a very good fixative, but tissues do not always stain well after being fixed in it. The tissues should be allowed to remain in the fluid for from two to twenty-four hours, according to size.

They will, when fixed, be found to be quite solid. They should then be removed and washed in running water over night and preserved in 80 per cent. alcohol.

FORMALIN.

Aqueous solutions of commercial formaldehyde may be used in from two to ten per cent. strength. Fixation is fairly good.

For the eye this is the best agent at our command. It must be used in 10 per cent. solution and a small nick should be made in one side of the globe to allow the fluid to penetrate the vitreous. *Alcohol must never be used for preserving eyes.*

